# Effect of the Interplay between Polymer–Filler and Filler–Filler Interactions on the Conductivity of a Filled Diblock Copolymer System

**DOI:** 10.3390/polym16010104

**Published:** 2023-12-29

**Authors:** A. I. Chervanyov

**Affiliations:** Institute of Theoretical Physics, University of Münster, 48149 Münster, Germany; chervany@uni-muenster.de

**Keywords:** diblock copolymers, filler, conductivity

## Abstract

We investigate the relative roles of the involved interactions and micro-phase morphology in the formation of the conductive filler network in an insulating diblock copolymer (DBC) system. By incorporating the filler immersion energy obtained by means of the phase-field model of the DBC into the Monte Carlo simulation of the filler system, we determined the equilibrium distribution of fillers in the DBC that assumes the lamellar or cylindrical (hexagonal) morphology. Furthermore, we used the resistor network model to calculate the conductivity of the simulated filler system. The obtained results essentially depend on the complicated interplay of the following three factors: (i) Geometry of the DBC micro-phase, in which fillers are preferentially localized; (ii) difference between the affinities of fillers for dissimilar copolymer blocks; (iii) interaction between fillers. The localization of fillers in the cylindrical DBC micro-phase has been found to most effectively promote the conductivity of the composite. The effect of the repulsive and attractive interactions between fillers on the conductivity of the filled DBC has been studied in detail. It is quantitatively demonstrated that this effect has different significance in the cases when the fillers are preferentially localized in the majority and minority micro-phases of the cylindrical DBC morphology.

## 1. Introduction

The possibility to control the preferential localization of fillers by changing the morphology of the host diblock copolymer system (DBC), well known from experiment [1,2,3] and theory [4,5,6,7,8,9], opens a wide perspective to the design of smart DBC-based composites. In contrast to chemically homogeneous polymer systems, the DBC can be used to “lock” fillers to their geometrically well-defined micro-phase domains, thus changing the properties of this system locally. One not yet properly explored application area of the unique properties of the filled DBC is the use of the described DBC morphology-controlled organization of fillers to direct the electrical response of DBC-based composites. This way of tailoring the electrical properties of soft composite materials can provide a superior alternative to the use of less convenient mechanical external stimuli [10,11,12,13] or shear [14]. Unlike these technically complicated methods, the desirable localization of fillers in the DBC can be achieved by directing the filler system obliquely, by external-stimuli-driven alteration of the morphology of a host DBC system.

In order to make the described prospect possible, one needs to have detailed quantitative understanding of which specific factors can be used most effectively, and in which combination, to precisely control the localization of fillers in the DBC. One important factor that determines the localization of fillers in a host DBC system, well known [1,15] experimentally, is the morphology of this system. Even so, it is qualitatively clear that the same DBC morphology can have varying effects on the filler localization. This effect depends, in particular, on the relative affinity of fillers for dissimilar copolymer blocks and the interaction between fillers. Previously, we have separately studied these two effects on the formation of the conductive filler network in the simplest lamellar morphology of the DBC [16,17]. In the present work, we essentially extend this study by investigating the complicated interplay between the effects caused by the filler–filler and filler–polymer interactions in a filled DBC system. We concentrate on the case of the cylindrical (hexagonal) morphology of the DBC as a host polymer matrix for conductive fillers. Note that this morphology can be experimentally realized both in a bulk DBC phase [18,19,20,21,22,23] and in thin DBC films [24]. It is also known [15] experimentally that the cylindrical morphology of the DBC can be maintained for relatively high nano-particle loads. The cylindrical morphology of the DBC, therefore, can be used as a reliable host polymer matrix that facilitates the formation of the conductive filler network. Moreover, by applying the developed approach, we quantitatively demonstrate that this morphology is a more promising candidate for using in the described electrical applications than its lamellar counterpart. In particular, we look into the relative roles of the filler–filler interactions and filler affinities for dissimilar copolymer blocks in promoting or suppressing the conductivity of the filler network formed in the cylindrical DBC morphology. One of the main objectives of this study is to prove that the effects caused by these interactions can be used as convenient tools to control the conductivity of the DBC-based composites.

In the part regarding the determination of the preferential localization of fillers in the DBC, the present approach relies on the continuum phase field model of the micro-phase-separated DBC coupled with the Monte Carlo (MC) simulation of the immersed filler system. Looking from the angle of the application to the performed conductivity calculation, the proposed model formulation has a number of advantages relative to the previous theories of filled polymer systems. In particular, our approach avoids using the continuum field approximation that relies on the thermodynamic average of the filler positions in the DBC, which is adopted in the Density Functional-Self-Consistent Field Theory (DFT-SCFT) [4,25]. We also do not use any artificial models for hard fillers, e.g., somewhat artificial “tagged function” representation of fillers adopted in [26]. Although being undoubtedly useful for the evaluation of the compositional structure of filled DBC systems, the above approaches are not suitable for the conductivity calculation performed in the present work. Our approach describes realistic finite-size fillers, thus making it possible to consistently calculate, based on the rigorous phase-field model, the energy of immersion of these fillers in the DBC. This feature is of key importance for the consistent formulation of the developed resistor network model used for the conductivity calculation in the present work.

Yet another superior feature of the developed approach is its ability to properly describe the effect of the volume excluded by the fillers to copolymers, overlooked in the above previous work. As is demonstrated in our present and previous work in [16,17], this osmotic effect, along with the interaction between fillers, is critical for predicting the localization of fillers in the DBC. In particular, the mentioned excluded volume effect explains the interfacial localization of neutral fillers observed in experiments [1]. Finally, the present phase-field-based approach circumvents significant technical limitations of the conceptually close, but more elaborate hybrid method [6]. Let us recall, this conceptually perspective method relies on performing the full SCFT calculation for the DBC system at every time step of the Brownian dynamic simulation for fillers immersed in this system. An increased computational demand, imposed by the described cumbersome computational procedure, can not only limit [27] the numerical accuracy of these calculations, but also impose limitations on the size of the simulated DBC system and number of fillers necessary for a quality conductivity calculation. Being more coarse-grained, the present method makes it possible to consider a whole variety of the compositions, morphologies and sizes of DBC–particle composites, while providing a sufficient accuracy at relatively low computational demand.

This paper is organized as follows. In Section 2.1, we use the phase-field model to derive the immersion energy of a spherical filler immersed in the micro-phase-separated DBC system. Section 2.2 is devoted to the prediction of the preferential localization of fillers in the micro-phases of the DBC for a given set of parameters that describe these fillers and DBC system. The obtained results are used in Section 2.3 to calculate the conductivity of a filled DBC system. In Section 3, we analyze the obtained simulation results and deduce the main factors affecting the conductivity of the composite. Section 4 details the conclusions and outlook.

## 2. Theory

### 2.1. Immersion Energy of a Filler in an Incompressible DBC System

Following our previous work in [9,16,17], we describe the compositional structure of the DBC system by a singe order parameter η. η varies between two limiting values 1 and −1 that correspond to the pure polymer phases *A* and *B*, respectively. The thermodynamic state of this system is described by the grand potential Ω, which is written as a functional of η, and reduced external fields $\phi_{A,B}=\beta \left(\mu_{A,B}-w_{A,B}\right)$. Here, $\mu_{A}$ ($\mu_{B}$) is the per-monomer chemical potential of block *A* (*B*) and $w_{A}$ ($w_{B}$) is the external potential that acts on the monomers comprising this block. As is shown in what follows, the introduced external potentials $w_{A}$ and $w_{B}$ can be used to describe the effect of fillers on the thermodynamic state of a DBC system. Grand potential Ω can be written in terms of the introduced coordinate($\vec{r}$)-dependent fields $\phi_{A,B}\left(\vec{r}\right)$ and $\eta (\vec{r})$, as follows:

(1) $$\begin{array}{c} \beta \Omega =-\frac{\rho R_{G}^{2}}{16Nf(1-f)}\int [\eta \left(\vec{r}\right)\nabla^{2}\eta \left(\vec{r}\right)+\xi^{-2}\eta {\left(\vec{r}\right)}^{2}\left(1-\frac{\eta {\left(\vec{r}\right)}^{2}}{2}\right)-\\ \frac{\lambda }{4\pi \xi^{4}}\left(\eta \left(\vec{r}\right)+\Delta f\right)\int {\left|\vec{r}-{\vec{r}}_{1}\right|}^{-1}\left(\eta \left({\vec{r}}_{1}\right)+\Delta f\right)d^{3}r_{1}]d^{3}r-\frac{\rho }{2}\int \eta \left(\vec{r}\right)\left(\phi_{A}-\phi_{B}\right)d^{3}r. \end{array}$$

Here, $\beta ={\left(kT\right)}^{-1}$ is the reciprocal temperature, *k* is the Boltzmann constant, *T* is the absolute temperature, *N* is the polymerization degree of copolymers, *f* is the diblock composition parameter, so that Nf and N(1−f) are the fractions of *A* and *B* monomers in each copolymer molecule, respectively, Δf=1−2f is the composition asymmetry parameter, ρ is the total density of the DBC system, $R_{G}$ is the gyration radius of copolymers, $\lambda =3\xi^{4}/R_{G}^{4}f\left(1-f\right)$, α≡χN is the segregation parameter, χ is the Flory–Huggins parameter that describes the interaction between dissimilar copolymer blocks. The second and third terms in the r.h.s. of Equation (1), which contain correlation length $\xi =R_{G}/\sqrt{4f(1-f)\alpha -o/(f(1-f))}$, describe the short-ranged and long-ranged correlations in a DBC system. The short-ranged correlations are described by the first two terms ∼$\eta^{2}\left(1-\frac{\eta^{2}}{2}\right)$ in the Ginzburg–Landau expansion of the DBC free energy in local order parameter η. The long-ranged correlations are described by the third integral term in the r.h.s. of Equation (1), which is proportional to λ. This term has been first derived by Ohta and Kawasaki in [28] to describe the effect of the connectivity of dissimilar blocks in a copolymer molecule on its structure factor. Fitting parameter *o* that enters correlation length ξ is determined for each DBC morphology by the fitting method described in [28]. For the symmetric case f=0.5, *o* has been found to amount to 0.9.

The density structure of a DBC system is described by the Lagrange equation for η obtained by the minimization of Ω for given values of chemical potentials $\mu_{A,B}$ that are set constant throughout the system. This equation reads as

(2) $$\begin{array}{c} \nabla^{2}\eta \left(\vec{r}\right)+\xi^{-2}\eta \left(\vec{r}\right)\left(1-\eta {\left(\vec{r}\right)}^{2}\right)-{\left(4\pi \right)}^{-1}\xi^{-4}\lambda \int {\left|\vec{r}-{\vec{r}}_{1}\right|}^{-1}\left(\eta \left({\vec{r}}_{1}\right)+\Delta f\right)d^{3}r_{1}+\\ 4NR_{G}^{-2}f\left(1-f\right)\left(\phi_{A}\left(\vec{r}\right)-\phi_{B}\left(\vec{r}\right)\right)=0. \end{array}$$

In the absence of the last term in its left-hand side (l.h.s.), the above equation describes the compositional structure of an incompressible DBC system. Note that, for a given composition *f*, this structure is fully determined by a single parameter λ. Decreasing λ below a certain threshold value $\lambda_{T}$ results in the periodic solutions $\eta_{0}$ of Equation (2), which describe the micro-phase-separated DBC system. For instance, in the case of pure (unfilled) symmetric DBC f=0.5, $\lambda_{T}$ is known [17] to amount to 0.25. The presence of external fields $w_{A,B}$, describing the effect of fillers on the DBC compositional structure, changes a simple periodic dependence of η. In this case, the exact solution of Equation (2) for a many-filler system becomes prohibitively complicated. For the tractability of the mathematical development, in the present work we resort to the simplified description of the polymer–filler interactions described below.

In the frameworks of the proposed model, the interaction of fillers with the DBC is described by the potential of the form

(3) $$w_{A,B}=-\rho^{-1}\epsilon_{A,B}\delta \left(r-R\right),$$

where δ(r) is the Dirac delta function, *r* is the distance to the center of a filler and *R* is the filler radius. The coefficient $\epsilon_{A}$ ($\epsilon_{B}$) of the delta function is the adhesion energy per unit area of copolymer block *A* (*B*) at the filler surface. ϵ is experimentally known [29,30] for most of the practically important polymer–filler pairs. Typically, $\epsilon_{A,B}$ is of the order of 10–50 mJ/m^2^. Physically, Equation (3) defines the potential of the weak adsorption interaction between fillers and polymers. This potential is assumed to have a range that is much smaller than the other characteristic lengths in the system (e.g., *R* and ξ). In addition, it is assumed that this weak adsorption interaction does not essentially affect the compositional structure of the incompressible DBC on the scale of correlation length ξ.

The defined polymer–filler interaction potential makes it possible to derive an explicit expression for the excess grand potential δΩ, caused by the presence of fillers. δΩ quantifies the minimal work required to reversibly immerse a filler into the DBC system at thermodynamic equilibrium. By its definition, δΩ can be obtained as the difference between the grand potential $\Omega [\eta \left(\vec{r}\right)]$ with non-zero potentials $w_{A,B}$ and its potential-free counterpart $\Omega [\eta_{0}\left(\vec{r}\right)]$. By making use of Equation (2) and the expressions for potentials $w_{A,B}$ given by Equation (3), one finds

(4) $$\beta \delta \Omega =-\frac{R^{2}\beta \epsilon }{4}\int \eta_{0}\left(\vec{r}+R\vec{n}\right)d\vec{n}+\frac{\rho R_{G}^{2}}{32Nf\left(1-f\right)\xi^{2}}\int_{V_{R}}\eta_{0}{\left(\vec{r}\right)}^{4}d^{3}r,$$

where $\vec{n}$ is the unit vector directed from the center of the filler to its surface point, $\epsilon =\epsilon_{A}-\epsilon_{B}$ quantifies the difference between the reduced enthalpies of the adsorption interactions between the fillers and the dissimilar copolymer blocks. Hereafter, ϵ is termed “affinity contrast”. The integration in the second term in the r.h.s. of Equation (4) is over the volume $V_{R}$ of a filler. This term, therefore, describes the excluded volume (osmotic) effect of fillers on the thermodynamic state of the DBC. The described osmotic effect stems from the fact that the copolymers are expelled from the space occupied by fillers. This effect promotes the localization of fillers at the interfaces between the DBC micro-phases, where these fillers screen the unfavorable contacts among dissimilar copolymer blocks. The first term in the r.h.s. of Equation (4), containing the integral over the surface of a filler, describes the adsorption interactions between the fillers and polymers. This term, therefore, favors the localization of fillers in the polymer phase that has a larger affinity for them. Relative significance of the described excluded volume and surface terms determines the preferential localization of fillers in a DBC system.

### 2.2. Localization of Fillers in Spatially Non-Uniform DBC

According to the Widom theorem [31], the probability to find a filler in a given location of a spatially non-uniform DBC system is proportional to exp(−βδΩ), where δΩ is the minimal work required for the reversible insertion of a filler into this system under given thermodynamic conditions. For the considered filled DBC system, this work is quantified by the excess grand potential given by Equation (4). As has been explained in Section 2.1, δΩ depends on the location of a filler in a spatially non-uniform DBC system. The probability to find a filler in a given location, therefore, depends on the composition of the DBC in the vicinity of this location. In addition to the above described factor, the probability of a given location of a filler in the DBC is affected by the concentration of other fillers near this location. This effect arises from the interactions between fillers that have been described by cumulative pair potential $U(\vec{r})$. As is shown in the next section, depending on the sign and strength of these interactions, the fillers tend to be distributed more diffusively, or, on the contrary, gather to form dense clusters.

To quantitatively elucidate the effect of the above two factors on the localization of fillers in a DBC system, we made use of the standard Metropolis algorithm implemented in the performed lattice Monte Carlo (MC) simulations [32]. Let us recall, this algorithm aims at determining the equilibrium filler distribution by iteratively selecting the distributions with the smallest total energy of the filler system. The calculation of the immersion part of this energy for the distributions of fillers, generated by the MC simulation runs, was performed in two steps. In the first step, we determined the morphological structure of the DBC by solving Equation (2) for $\eta (\vec{r})$ for the case of pure DBC corresponding to $\phi_{A}=\phi_{B}=0$. This solution has been obtained by the iso-geometric finite element method [33] for selected values of control parameter λ and fixed DBC composition f=0.45. This composition value corresponds to the lamellar or cylindrical morphology of the DBC, depending on the value of the control parameter λ. Hereafter, λ is termed “segregation”, for the sake of brevity. The immersion energy of fillers is calculated by the numerical integration of the obtained solution for η for all the filler distributions generated by MC. Technically, this calculation is performed by numerically evaluating the integrals in Equation (4). Affinity contrast ϵ in the pre-factor of the surface integral in this formula is fixed in each simulation set, and it varies in different sets. This variation mimics the effect of the filler affinity contrast for dissimilar DBC blocks, which most significantly affects the localization of fillers. This procedure is described in Section 3 in detail.

The pair interaction between fillers is modeled by position-independent (attractive or repulsive) potential *U* that acts between the fillers occupying the nearest-neighboring sites of the cubic lattice used in the simulations. The period of this lattice is set equal to the radius of the fillers, which presents a smallest characteristic length in the system. Note that this setting minimizes the influence of the selected lattice geometry on the simulation results. The selected positive (repulsive) and negative (attractive) values of *U* are used in different simulation sets to elucidate the effect of the inter-filler interaction on the formation of the filler-conductive clusters in different DBC morphologies.

Overall, the performed simulation procedure is designed for determining the most probable equilibrium localization of fillers immersed in a DBC system that assumes a given morphology. Recall that this morphology is fully determined by the control parameters λ and *f*. The described localization of fillers in DBC micro-phases has been altered by changing the affinity contrast ϵ and inter-filler interaction potential *U* in different simulation sets. The output of the simulation is given in the form of the spatial coordinates of fillers corresponding to their equilibrium distribution, obtained after $10^{6}$ runs of the simulation in each set. The corresponding conductance of the composite is given along with each calculated filler distribution (see Section 2.3). The standard [32] 95%-confidence interval ΔE of the total energy of the filler system, obtained in each set of the simulations, is determined to monitor the accuracy of the simulation for a given number of runs. The average value of ΔE, obtained for segregation λ=0.2 and composition f=0.45, evaluates to $9.0\times 10^{-3}$ kT. This relatively small ΔE corroborates the high accuracy of the performed simulations.

### 2.3. Effect of Filler Localization on the Electrical Conductivity of a Filled DBC System

The equilibrium distribution of fillers in DBC system, obtained by the MC simulations described in Section 2.2, is used to calculate the electrical conductivity of this system. We consider that constant voltage *W* is imposed across the layer of the composite placed between the plane electrodes. These electrodes are oriented perpendicular to the axes of the cylindrical or lamellar DBC micro-phases. The DBC is assumed to be completely insulating, so the role of this compositionally inhomogeneous polymer matrix reduces to facilitating the formation of the conductive network of fillers. As is shown in what follows, at a sufficient volume fractions of fillers, the continuous percolating filler network that connects the electrodes and conducts the electrical current, is formed. The minimal filler volume fraction that is sufficient to provide the described conductive network, depends on the morphology of the DBC system and the filler affinities for DBC blocks.

The lattice model used for the calculation of the equilibrium distribution of fillers described in Section 2.2 can be naturally incorporated into the calculation of the composite conductivity described below. As a basic assumption of the present conductivity model, we consider that the fillers, occupying neighboring sites of the lattice, form a conductive bond having fixed conductivity σ. For the purposes of the conductivity calculation, this lattice can be, therefore, represented as a resistor network. This network is composed of the conductive bonds formed by neighboring fillers. The conductivity of the described resistor network has been calculated by making use of the Kirchhoff’s relations written for each node defined by the lattice coordinates l,i,j. These relations read as

(5) $$\begin{array}{c} U_{i,j}^{l+1}=U_{i,j}^{l}+Z_{i,j}^{l-1}I_{i,j}^{l+1},\\ I_{i,j}^{l+1}=I_{i,j}^{l}+X_{i,j}^{l}\left(U_{i,j}^{l}-U_{i+1,j}^{l}\right)+X_{i-1,j}^{l}\left(U_{i,j}^{l}-U_{i-1,j}^{l}\right)+\\ Y_{i,j}^{l}\left(U_{i,j}^{l}-U_{i,j+1}^{l}\right)+Y_{i,j-1}^{l}\left(U_{i,j}^{l}-U_{i,j-1}^{l}\right), \end{array}$$

where $I_{i,j}^{l}$ and $U_{i,j}^{l}$ are the current and voltage at node (l,i,j). Here, $X_{i,j}^{l}$, $Y_{i,j}^{l}$ and $Z_{i,j}^{l}$ denote the bonds corresponding to the unit vectors of the standard xyz orthogonal Cartesian system with the origin at l,i,j and the xy plane parallel to the electrodes. Note that $Z_{i,j}^{l}$ is normal and $X_{i,j}^{l}$, $Y_{i,j}^{l}$ are parallel to the electrodes. Each of the $X_{i,j}^{l}$, $Y_{i,j}^{l}$ and $Z_{i,j}^{l}$ bonds is assigned the conductivity σ or 0, depending on the location of fillers in the nodes connected by this bond. Recall that the location of fillers in given lattice nodes is specific to each distribution of fillers obtained from the MC simulation runs, as described in Section 2.2.

The iteration relations given by Equation (5) have been solved numerically for each filler distribution, generated by the MC simulation runs, in the presence of boundary conditions $U_{ij}^{0}=0$ and $U_{ij}^{L}=W$. These conditions fix the constant voltages at the negative (l=0) and positive (l=L) electrodes. The obtained solution has been then used to calculate the conductance *S* of the composite by applying Ohm’s law $S=\sum_{i,j}\left(I_{i,j}^{L}-I_{i,j}^{0}\right)/W$.

The obtained result for conductance *S* of the composite is proportional to the elementary conductance σ of the bond formed by neighboring pair of fillers. It is therefore instructive to measure *S* relative to the conductance $S_{0}$ of the lattice fully occupied by fillers. In the framework of the developed model, the conductance of this lattice corresponds to that of a pure filler material known in most practical cases. Note that the reduced conductance $S/S_{0}$ does not depend on σ. $S/S_{0}$ can be thus obtained from the above described simulations, without any additional model evaluations of σ that can cause model-specific uncertainties.

## 3. Results and Discussion

The described simulation procedure makes it possible to thoroughly investigate the distribution of fillers in the micro-phase separated DBC system, as well as the conductivity of this system. In all the simulation sets, the radius of fillers is set equal to 10 nm. The variable control parameters used to alter the distribution of fillers for a given DBC morphology are the affinity contrast ϵ of fillers for dissimilar copolymer blocks and the interaction energy *U* between fillers. The DBC morphology, in turn, is altered by changing the segregation parameter λ. Recall that the second parameter affecting the DBC morphology, i.e., the DBC composition *f*, is set equal to 0.45 corresponding to a slightly asymmetric DBC composition. For this composition, the simulations have shown the lamellar and cylindrical morphologies of a pure DBC system in the intervals λ∈[0,0.193] and λ∈(0.193,0.238], respectively. The order–order transition between the lamellar and cylindrical morphologies were found to occur at λ=0.193 and the order–disorder transition at λ=0.238.

**Figure 1:**
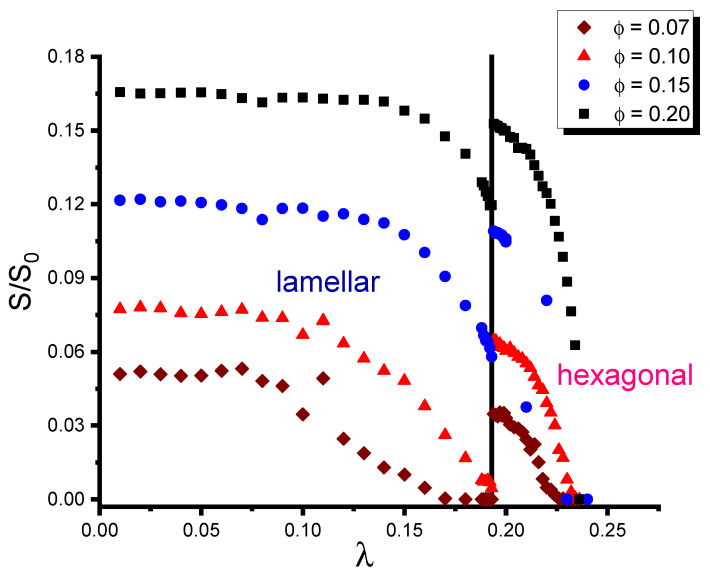
Conductivity of the micro-phase-separated asymmetric DBC system (f=0.45) as a function of segregation parameter λ for selected volume fractions of fillers φ.

Changing the morphology of the host DBC system results in changing the location of fillers in this system, thus affecting its conductivity. This effect is illustrated in Figure 1. This figure shows the reduced conductivity calculated by the method described in Section 2.3. Each point in this figure illustrates a single simulation set performed for the corresponding values of filler volume fraction φ and segregation λ. Affinity contrast ϵ and reduced interaction energy βU between fillers are set equal to 5 mJ/m^2^ and −1, respectively, for all the simulation sets shown in Figure 1. The used value of the interaction energy corresponds to a weak Van der Waals attraction between fillers, which does not significantly affect their distribution in the DBC. The DBC morphologies were generated by incrementally increasing λ in the interval [0,0.24] by keeping f=0.45 constant. The composites containing four different volume fractions φ=0.07,0.10,0.15,0.20 of fillers were modeled in four respective sequences of the simulation sets.

As can be seen from Figure 1, the results for all the investigated volume fractions of fillers show similar trends. At smaller values of segregation λ<∼0.1 corresponding to the strong segregation regime of the DBC, the conductivity of the composite is at its maximum and it is almost independent of λ. This observation is explained by the fact that, at the strong segregation, the DBC forms the clear-cut lamellar domains with narrow interfaces between them. These conditions are most favorable for the localization of fillers in the selective polymer phase (*A*) having larger affinity for these fillers. For any given affinity contrast ϵ and filler fraction φ, the filler concentration in the selective phase saturates at certain $\lambda_{S}∼0.1$, specific to each φ and ϵ. This effect results in the maximum conductance of the composite for $\lambda <\lambda_{S}$, specific to each volume fraction of fillers.

With increasing λ above $\lambda_{S}$, up to the order–order transition point, the segregation of the micro-phase-separated DBC diminishes. Respectively, the distribution of fillers in the lamellae becomes more diffuse, as the fillers tend to localize not only in the selective DBC micro-phases, but also at the interfaces between them. Wider interfaces formed at larger λ, therefore, promote the depletion of the local density of fillers in the DBC. This effect, in turn, results in the reduction in the conductivity of the composite.

The most remarkable feature, observed in Figure 1, is the spike in the conductivity that occurs at $\lambda_{OOT}=0.193$ for all the studied volume fractions of fillers. By direct comparison with the DBC morphologies obtained in the simulations for the corresponding values of λ, one finds that this spike is associated with the order–order transition (OOT) between the lamellar and cylindrical micro-phases of DBC. At close values of λ in the vicinity of the OOT point, the conductivity of the filler network formed in the cylindrical DBC micro-phase was found to be several times larger than that of its counterpart formed in the lamellar micro-phase. The magnitude of the described conductivity jump upon crossing $\lambda_{OOT}$ only slightly depends on the volume fraction of fillers, being most pronounced at the moderate filler volume fraction of ∼0.1. This effect is explained by different spatial organization of fillers in the cylindrical and lamellar micro-phases of the DBC. The fillers that are trapped in the concise cylinders of the selective *A*-phase by the adhesion force form less branched conductive clusters than their counterparts located in the lamellar phase. These clusters, forming shorter conductive paths, promote the observed larger conductivity of the composite that assumes the cylindrical morphology.

**Figure 2:**
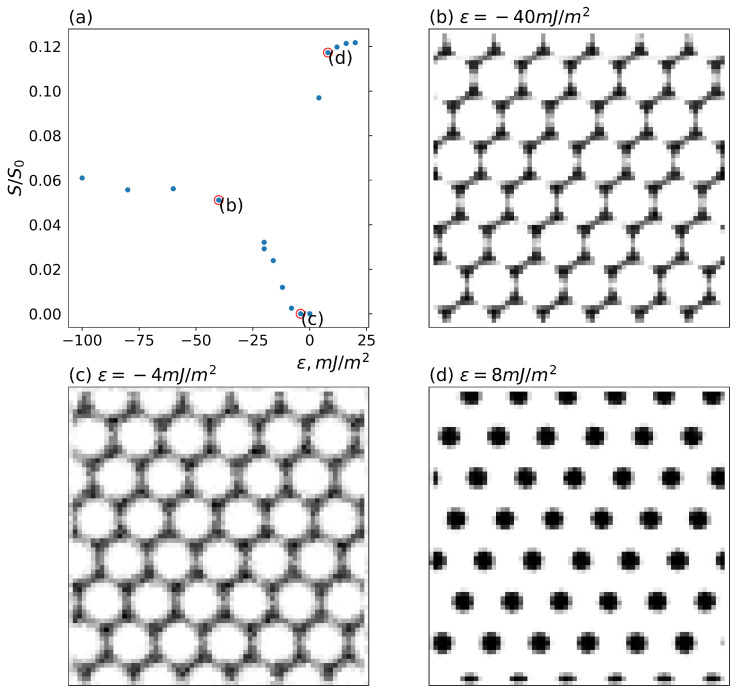
Dependence of the conductivity of the micro-phase-separated asymmetric DBC system (f=0.45) that assumes the cylindrical morphology for different localizations of fillers in DBC micro-phases. Segregation λ, volume fraction of fillers φ and reduced inter-filler interaction energy βU are set equal to 0.2, 0.1 and −1.0, respectively. (**a**) Conductivity as a function of the filler affinity contrast for dissimilar copolymer blocks ϵ. (**b**–**d**) Localization of fillers for selected values of ϵ: (**b**) ϵ = −40 mJ/m^2^, (**c**) ϵ = −4 mJ/m^2^, (**d**) ϵ = 8 mJ/m^2^. See explanation in the text.

**Figure 3:**
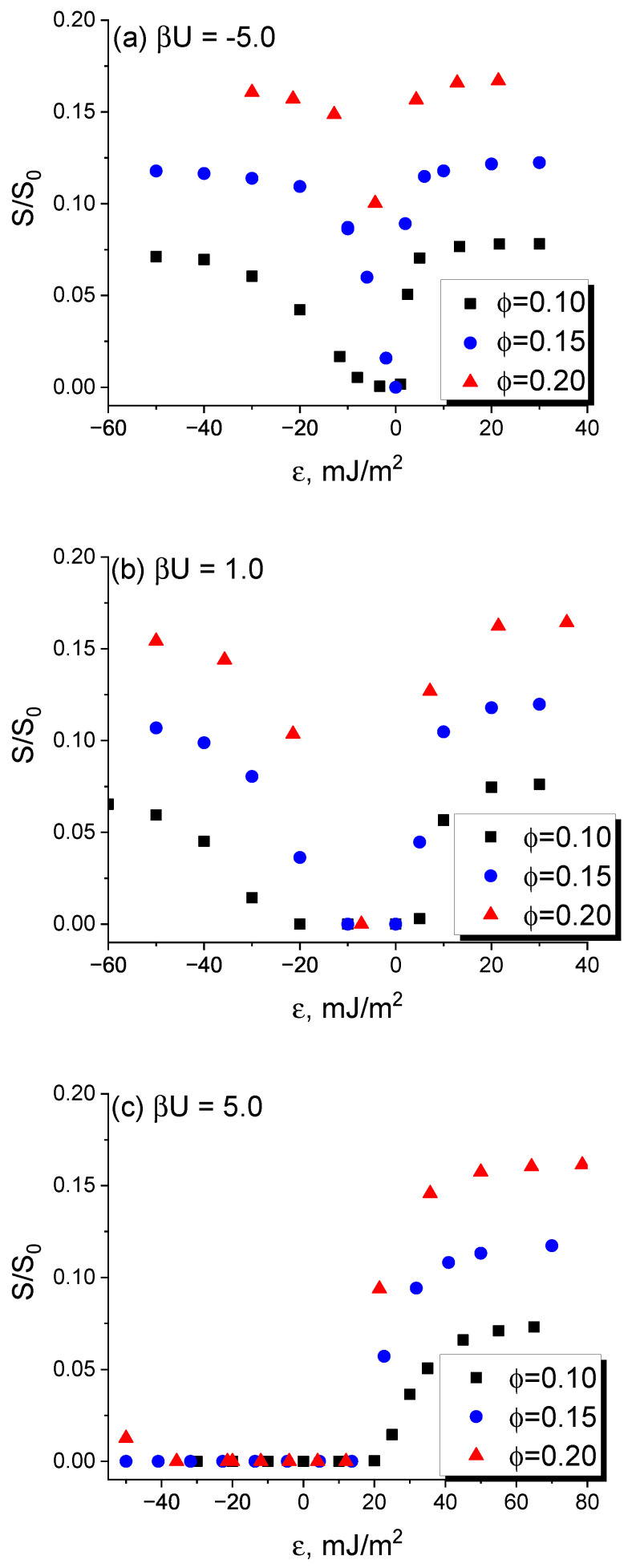
Conductivity of the micro-phase-separated asymmetric DBC system (f=0.45) that assumes cylindrical morphology as a function of the filler affinity contrast for dissimilar copolymer blocks ϵ for selected inter-filler interaction energies βU. Segregation λ and volume fraction of fillers φ are set equal to 0.2 and 0.15, respectively. (**a**) βU=−5.0. (**b**) βU=1.0. (**c**) βU=5.0. See explanation in the text.

Since the cylindrical morphology of the DBC is found to be more efficient for the formation of the filler conductive network, it is instructive to investigate the distribution of fillers in this morphology in relation to the conductivity of the composite. In the next simulation round, we have used the cylindrical morphology generated from the solution of Equation (2) for the fixed value λ=0.2. Note that this value of λ lies slightly above the point of the order–order transition from the lamellar to cylindrical DBC morphology. The three volume fractions φ=0.1, 0.15 and 0.20 of the fillers are studied. The localization of the fillers was directed by varying affinity contrast ϵ and inter-filler interaction energy *U*. The obtained results are illustrated in Figure 2 and Figure 3.

Figure 2 shows the effect of altering the affinity contrast ϵ on the localization of fillers and the composite conductivity. In the simulation round illustrated by Figure 2, the volume fraction of the fillers has been set equal to 0.15. The reduced interaction between fillers is fixed to βU=−1 corresponding to weak (e.g., Van der Waals, depletion) attraction between fillers. Subplots (b)–(d) in this Figure illustrate the volume fraction of fillers averaged along the direction perpendicular to the cylinders of the hexagonal phase. Subplot (a) shows the conductivities of the described filler system for different affinity contrasts ϵ of the fillers for dissimilar copolymer blocks. The marked points of the scatter in subplot (a) correspond to the respective filler distributions illustrated in subplots (b)–(d).

Note that affinity contrast ϵ has different signs. The positive values of ϵ correspond to the larger affinity of the minority cylindrical micro-phase *A* for the fillers. As should be expected, for sufficiently large positive ϵ (see subplot (d) in Figure 2), the fillers are mainly localized within the cylinders of the *A*-micro-phase. The reduced composite conductivity $S/S_{0}$ defined in Section 2.3, corresponding to this case (see point (d) in the subplot (a) of Figure 2), reaches its maximum value of ∼0.12.

At sufficiently large negative values of ϵ, the fillers were found to be localized in the majority *B*-micro-phase. Interestingly, at sufficiently large total volume fraction of fillers, the described localization of the fillers in the *B*-micro-phase proves to provide for the formation of the percolating conductive filler clusters. Since the distribution of fillers in the majority *B*-micro-phase is more diffuse, the resulting conductivity at its maximum is approximately two times smaller than that observed for the localization of fillers in the cylindrical *A*-micro-phase (see point (b) in the subplot (a) of Figure 2). Moreover, the conductivity of the filler system, having larger affinity for the *A*-micro-phase, shows a much steeper increase with increasing affinity contrast |ϵ|, as compared to its counterpart observed for the filler system localized in the majority *B*-micro-phase. While the conductivity of the filler network localized in the cylinders of the *A*-micro-phase reaches its maximum at ϵ∼ 20 mJ/m^2^, the maximum conductivity of the filler network in the *B*-micro-phase is achieved at ϵ∼−60 mJ/m^2^. This brings us to the conclusion that the localization of fillers in the cylinders of the minority *A*-micro-phase can be most efficiently used for the formation of a percolating network.

For small affinity contrasts |ϵ|, the role of the interfacial localization of fillers increases. Recall that, in this case, the fillers tend to localize at the interfaces between the micro-phases *A* and *B*, to screen the unfavorable interactions between the dissimilar copolymer blocks. Mathematically, this effect is described by the increased role of the osmotic term given by the second term in the r.h.s. of Equation (4). The described effect is especially pronounced for the case when the affinity of the fillers is slightly larger for the majority *B*-micro-phase (see subplot (c) in Figure 2). In this case of small negative ϵ, the fillers are distributed more diffusively throughout the larger volume, composed of the *B*-micro-phases and *A*-*B* interfaces. The described more diffuse distribution of fillers results in decreasing the conductivity of the composite (see point (c) in the subplot (a) of Figure 2) relative to the case of larger |ϵ|. The described decrease in the conductivity with decreasing |ϵ| is attributed to an increase in the filler network branching and the reduction in the long-ranged percolative paths in a larger volume, available to the localized fillers.

To systematically investigate the effect of the interaction between fillers on their localization in the DBC and the conductivity of the composite, we have extended the simulation described in the first part of this section over the cases of filler volume fractions φ=0.10, 0.15 and 0.20 and the reduced filler–filler interaction energies βU=−5.0, 1.0 and 5.0. The results of these simulations are shown in Figure 3. The main trend that can be derived from the comparison of subplots (a)–(c) in Figure 3 is that the sign of the filler–filler interactions energy has a significant effect on the composite conductivity for ϵ<0. Recall that this case corresponds to a larger affinity of fillers for the majority *B*-micro-phase. Specifically, relatively strong repulsive interaction between fillers suppresses the conductivity. For the case βU=5.0, the conductivity completely vanishes for ϵ< ∼5 mJ/m^2^ at smaller filler volume fractions of 0.10, 0.15. For a larger filler fraction of 0.20, the non-zero conductivity is observed only at larger affinity contrasts ϵ<−50 mJ/m^2^. The same effect of the reduction in the composite conductivity caused by the repulsive interactions between fillers was observed also for ϵ<0, but it was found to be far less significant. One can therefore conclude that, in the considered case of the cylindrical morphology, the affinity contrast of fillers for dissimilar copolymer blocks has larger significance for the composite conductivity than the inter-filler interactions. A sufficiently strong repulsive interaction between fillers can suppress the conductivity of a filler system located in the majority *B*-micro-phase of the DBC, but it has a rather negligible effect when fillers are localized within the cylinders comprising the minority *A*-micro-phase.

## 4. Conclusions

The present work extends the method, previously developed [9,16,17] by the author, over the study of the relative roles of the filler affinity for copolymer blocks and the interaction between fillers in promoting the conductivity of a filled DBC system. Specifically, we focused on the study of the effect of the above two factors on the localization of fillers in the DBC-based composite that assumes cylindrical (hexagonal) morphology. Furthermore, we elucidated the relation between the investigated localization of fillers and the formation of the conductive filler network in this composite. In addition, we investigated the effect of the order–order transition between the lamellar and cylindrical morphologies of DBC on the conductivity of the filler network formed in the DBC system.

Technically, the present work has been performed in three consecutive stages. In the initial stage, we employed the finite element method to solve Equation (2) that describes the compositional structure of a filled DBC system in dependence on DBC segregation λ and composition *f*. The obtained solution was then used to deduce the immersion energy of a filler given by Equation (4) as a function of the position of this filler in a DBC system. The output of the described first stage was used in the second stage, where the standard Metropolis Monte Carlo simulation was employed to determine the equilibrium distribution of fillers in the micro-phase-separated DBC system. The simulated filler distribution was found to essentially depend on the composite morphology, filer–polymer and filler–filler interactions and volume fractions of the fillers. In the third stage, the simulated filler distribution was used to calculate the conductance of the filler network generated by the Monte Carlo simulations. As a main result of the described simulation procedure, the conductivity of the composite was obtained for the selected DBC morphologies, filler fractions and strengths of the interactions between the composite components.

The obtained results were used to thoroughly analyze the interplay between the two main influence factors, i.e., the difference between the filler affinities for dissimilar copolymer blocks and the interaction between fillers, in relevance to the conductivity of the composite. In the present work, this analysis focused on the composite that assumes cylindrical morphology. Our first important finding is that the cylindrical morphology of the DBC is more efficient, compared to the lamellar morphology, in facilitating the formation of the conductive filler network. This observation can be directly derived from Figure 1 by the comparison of the composite conductivities in the vicinity of the order–order transition. This comparison shows that the conductivity of the composite that assumes the cylindrical morphology is much larger than that of its lamellar morphology-based counterpart at the same DBC composition and close λ (temperatures). The second important finding is that the localization of fillers inside the cylinders of the DBC minority micro-phase provides for much larger conductivity of the composite relative to the filler localization in the majority micro-phase. In addition, the conductivity of the filler network localized in the minority micro-phase reaches its maximum at relatively low affinity contrast ∼20 mJ/m^2^ of fillers for dissimilar copolymer blocks. For the opposite case of the filler localization in the majority DBC micro-phase, the affinity contrast sufficient to provide for a maximum conductivity, in contrast, is relatively large (∼60 mJ/m^2^). The third main finding is that the effect of the interactions between fillers has a different significance depending on whether the minority or majority DBC micro-phase has a larger affinity for fillers. Specifically, a sufficiently large repulsive interaction between fillers has been found to suppress the conductivity of the composite containing fillers localized in the majority micro-phase. This observation is attributed to the fact that strong repulsive interactions promote more diffuse distribution of fillers in this micro-phase, thus preventing the formation of sufficiently long conductive clusters. Interestingly, the magnitude of this effect is not sufficient to suppress the conductivity of the filler network located inside the concise cylinders of the minority micro-phase under the same conditions. The attractive interactions between fillers has just the opposite effect on the formation of the conductive filler network located in the majority micro-phase. Specifically, these interactions have been found to enhance the composite conductivity. This effect is especially pronounced for moderate volume fraction of fillers φ=0.1. As can be seen from Figure 3, the conductivity of the filler network located in the cylinders of the minority micro-phase, in contrast, is only slightly affected by the interaction between fillers.

The present work elucidates the role of the polymer–filler and filler–filler interactions in promoting or suppressing the conductivity of a filled DBC system. By manipulating these interactions (e.g., by surface treatment of fillers or using appropriate polymer grafts that change the filler relative affinity for DBC blocks), one can achieve a desirable electrical response of the DBC-based composite. The theoretical analysis of the relevant effects, provided in the present work, can therefore pave the path toward designing DBC–nano-particle composites with controlled electrical response. These composites can be used, in particular, in such important industrial applications as soft sensors [13,34,35,36,37] and flexible electronics [38,39].

## Data Availability

Data are contained within the article.

## Abbreviations

The following abbreviations are used in this manuscript:

DBCs	diblock copolymers
MC	Monte Carlo
OOT	odrer–order transition
r.(l.)h.s.	right (left)-hand side
